# Impact of different levels of diastema and crowding on the precision of 3D-printed dental models: a comparative analysis using digital superimposition

**DOI:** 10.1590/2177-6709.31.1.e2624241.oar

**Published:** 2026-04-17

**Authors:** Farhad SALMANPOUR, Hasan CAMCI

**Affiliations:** 1 Afyonkarahisar Health Sciences University, Dental School, Department of Orthodontics (Afyonkarahisar, Türkiye).

**Keywords:** Digital orthodontics, 3D printing, Dimensional precision, Dental crowding, Diastema, Digital superimposition, Ortodontia digital, Impressão 3D, Precisão dimensional, Apinhamento dentário, Diastema, Sobreposição digital

## Abstract

**Introduction:**

This study aimed to evaluate the impact of varying degrees of anterior diastema and crowding on the precision of 3D-printed dental models using digital superimposition techniques.

**Materials and Methods:**

A digital maxillary arch model was modified in the anterior region (canine to canine) to simulate three levels of diastema (2.5 mm, 5 mm, 10 mm) and four levels of crowding (3 mm, 6 mm, 9 mm, 12 mm), along with an unmodified control. Eight digital models were fabricated using LCD 3D printing, with 15 prints per group. Printed models were scanned and superimposed onto their respective reference models using Geomagic Control X. Surface deviations were analyzed via minimum, maximum, root mean square (RMS), and average positive and negative values. One-way ANOVA and Tukey’s HSD *post-hoc* test were used for statistical evaluation.

**Results:**

Significant differences were observed among diastema groups (p < 0.05), with the 5 mm group showing the widest deviation range. RMS and average deviation values were highest in the 2.5 mm and 5 mm diastema conditions. In the crowding groups, significant deviations in minimum and maximum values occurred only in the 12 mm group (p < 0.001 and p < 0.008, respectively).

**Conclusion:**

Severe anterior crowding (12 mm) and mild to moderate diastemas (≤5 mm) significantly impair the precision of 3D-printed dental models. These results highlight the importance of assessing digital model fidelity in cases with anterior spacing or crowding, to ensure accurate diagnosis and appliance fabrication.

## RESUMO

**Introdução:**

Este estudo teve como objetivo avaliar, utilizando técnicas de sobreposição digital, o impacto de diferentes graus de diastema anterior e apinhamento na precisão de modelos dentários impressos em 3D.

**Material e Métodos:**

Um modelo digital da arcada superior foi modificado na região anterior (canino a canino) para simular três níveis de diastema (2,5 mm, 5 mm, 10 mm) e quatro níveis de apinhamento (3 mm, 6 mm, 9 mm, 12 mm), juntamente com um controle não modificado. Assim, oito modelos digitais foram fabricados utilizando impressão 3D LCD, com 15 impressões por grupo. Os modelos impressos foram escaneados e sobrepostos aos seus respectivos modelos de referência, utilizando o software Geomagic Control X. Os desvios de superfície foram analisados por meio dos valores mínimo, máximo, raiz quadrada média (RMS) e média dos valores positivos e negativos. Para avaliação estatística, foram utilizados os testes ANOVA de uma via e *post-hoc* HSD de Tukey.

**Resultados:**

Foram observadas diferenças significativas entre os grupos com diastema (p < 0,05), sendo que o grupo de 5 mm apresentou a maior amplitude de desvio. Os valores de RMS e desvio médio foram mais elevados nas condições de diastema de 2,5 mm e 5 mm. Nos grupos com apinhamento, desvios significativos nos valores mínimo e máximo ocorreram apenas no grupo de 12 mm (p < 0,001 e p < 0,008, respectivamente).

**Conclusão:**

O apinhamento anterior severo (12 mm) e os diastemas leves a moderados (≤5 mm) comprometem significativamente a precisão dos modelos dentários impressos em 3D. Esses resultados destacam a importância de se avaliar a fidelidade do modelo digital em casos de diastema anterior ou apinhamento, para garantir um diagnóstico preciso e a confecção adequada de aparelhos ortodônticos.

## INTRODUCTION

Three-dimensional (3D) printing technologies have become an integral part of modern orthodontic practice, facilitating the fabrication of diagnostic models, clear aligners, indirect bonding trays, and retainers within fully digital workflows. Among the available technologies, vat polymerization systems - such as stereolithography (SLA), digital light processing (DLP), and liquid crystal display (LCD) - are particularly well-suited for dental applications, due to their high resolution and ability to reproduce fine anatomical details.^1^ ^-^ ^3^

While printer-related variables such as resin composition, photopolymerization parameters, and layer thickness have been extensively studied for their impact on model accuracy, the role of intraoral anatomical variations has received comparatively little attention. Specifically, anterior dental crowding and diastema may compromise intraoral scan accuracy, introducing artifacts that propagate through the digital workflow and ultimately impair the dimensional fidelity of printed models.^4^ ^-^ ^6^ This is clinically relevant, as even minor geometric discrepancies can undermine appliance fit and reduce treatment efficacy.

In clear aligner therapy, for example, inaccurate capture of anterior spacing may result in incomplete diastema closure, leading to residual esthetic discrepancies at the conclusion of treatment. Similarly, inadequate representation of crowded dentitions can affect the programmed force vectors within aligner systems, diminishing tooth movement efficiency and prolonging treatment duration.^7^ ^-^ ^9^

The consequences of model inaccuracy are not limited to aligner-based protocols. In indirect bonding techniques - where brackets are positioned on printed models and transferred intraorally using customized trays - even subtle distortions may lead to clinically significant deviations in bracket placement. Such errors can alter planned tooth movement trajectories, necessitate re-bonding procedures, or increase the need for chairside adjustments. Likewise, inaccuracies in printed models used for retainer fabrication may result in suboptimal appliance fit, jeopardizing long-term post-treatment stability.^10^

Beyond biomechanical implications, reduced model accuracy can disrupt the overall clinical workflow by increasing adjustment appointments, elevating material costs, delaying treatment timelines, and diminishing patient satisfaction.^11^ As digital orthodontics continues to evolve, ensuring the fidelity of printed models remains critical for preserving treatment efficiency and quality of care.

Despite its clinical relevance, limited research has investigated how varying degrees of anterior spacing and crowding affect the accuracy of 3D-printed orthodontic models. Given the esthetic and functional significance of the anterior region, a more detailed understanding of these anatomical factors is essential for optimizing appliance fabrication and improving clinical outcomes.^12^ ^-^ ^14^

Therefore, the aim of this study was to assess the effect of different levels of anterior diastema and crowding on the dimensional accuracy of 3D-printed orthodontic models. The null hypothesis was that neither spacing nor crowding would significantly influence model accuracy.

## MATERIAL AND METHODS

A digital intraoral scan of a fully treated maxillary arch, free of carious lesions and restorations, was selected as the reference model for this study. Using digital setup procedures, seven modifications were applied to the anterior segment (canine to canine) to simulate varying levels of spacing and crowding: 2.5 mm diastema (0.5 mm between adjacent teeth), 5 mm diastema (1.0 mm), 10 mm diastema (2.0 mm), and crowding conditions of 3 mm, 6 mm, 9 mm, and 12 mm.

All virtual manipulations were carried out using OrthoAnalyzer software (3Shape, Copenhagen, Denmark). Within the segmentation module, anatomical boundaries of the canines and incisors were delineated using the software’s marker tool to enable automatic tooth segmentation. In cases where automatic segmentation proved insufficient, manual refinements were performed. Following segmentation, the anterior teeth were digitally isolated and repositioned to reflect the specified degrees of diastema and crowding.

**Figure 1:**
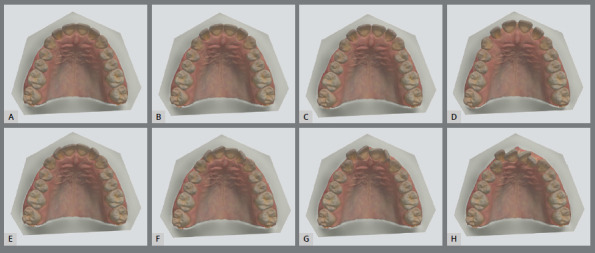
A) Main model, B) 2.5 mm diastema, C) 5 mm diastema, D) 10 mm diastema, E) 3 mm crowding, F) 6 mm crowding, G) 9 mm crowding, H) 12 mm crowding.

Crowding severity was categorized according to a modified version of Little’s Irregularity Index, resulting in four distinct groups: minimal (3 mm), moderate (6 mm), severe (9 mm), and very severe (12 mm).^14^ However, due to the absence of a universally accepted classification system for diastema severity in the literature, three spacing levels were defined, based on total interproximal gaps: 2.5 mm (0.5 mm between adjacent teeth), 5 mm (1.0 mm), and 10 mm (2.0 mm) (Fig. 1).

**Figure 2:**
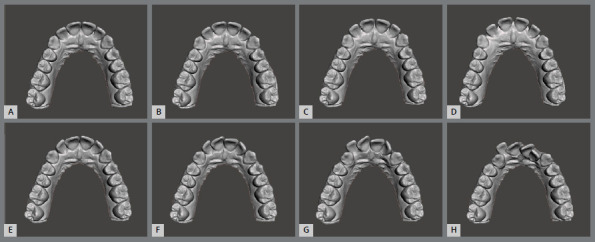
Models that have been optimized with the Meshmixer software before printing: **A)** Main model, **B)** 2.5 mm diastema, **C)** 5 mm diastema, **D)** 10 mm diastema, **E)**3 mm crowding, **F)** 6 mm crowding, **G)** 9 mm crowding, **H)** 12 mm crowding.

Sample size estimation was performed using G*Power software 3.1.9.7 (Heinrich Heine Universität, Düsseldorf, Germany), which indicated that 15 printed models per group were required to detect a moderate effect size (f = 0.36), with 80% statistical power and a significance level of 0.05.^13^ Each digital model was subsequently optimized for 3D printing and superimposition using Meshmixer software (version 3.1.373; Autodesk, San Rafael, CA, USA) (Fig. 2). Prior to printing, models were oriented at a 30° inclination, and support structures were generated in Chitubox software (version 1.9.5; Guangdong, China) according to the manufacturer’s guidelines. A layer thickness of 50 µm was selected for all prints. The models were fabricated using a Phrozen Sonic Mini 8K printer (Phrozen, Hsinchu City, Taiwan), with printer calibration conducted prior to each printing session. In total, 120 physical models (15 per group) were produced.

Following the printing process, residual uncured resin was removed using ultrasonic cleaning. Each model underwent two cycles of ultrasonic cleaning in 97% isopropyl alcohol, with each cycle lasting three minutes. The alcohol solution was refreshed before each session. After cleaning, models were air-dried for 30 seconds and then post-cured in a UV curing unit (Phrozen Anycubic Wash and Cure Plus; Hsinchu City, Taiwan) for 20 minutes using a 36-watt ultraviolet light source (Senertek, Izmir, Turkey), following the manufacturer’s instructions.^15^

After post-curing, all printed models were digitized using a TRIOS intraoral scanner (3Shape, Copenhagen, Denmark) by a single calibrated operator. Scanning was performed under standardized ambient lighting, monitored using a luxmeter to ensure consistent illumination.

**Figure 3:**
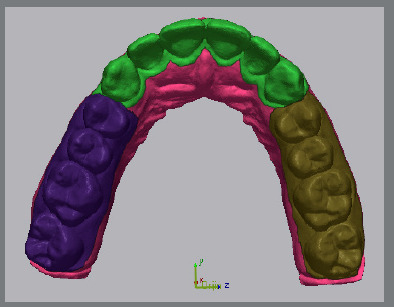
The models were digitally segmented into three regions: anterior (from canine to canine), left posterior (from the first premolar to the second molar), and right posterior (from the first premolar to the second molar). The teeth in the right and left posterior regions, which were not altered during the digital setup, served as reference points for the three-dimensional model superimposition.

For surface deviation analysis, both the reference (original) and scanned (experimental) digital models were imported into Geomagic Control X 2022 software (3D Systems, Rock Hill, SC, USA). Superimposition was performed using the Best-Fit alignment algorithm.^16^ The posterior teeth, which remained unaltered during the digital setup, were designated as the reference region for alignment. The region of interest - the anterior segment from canine to canine, including 1 mm apical to the cemento-enamel junction - was then isolated for surface deviation analysis (Fig. 3).

Surface deviations were quantified using the following parameters:

» Minimum (Min): The largest negative deviation, representing inward displacement relative to the reference model.
» Maximum (Max): The largest positive deviation, representing outward displacement relative to the reference model.
» Root Mean Square (RMS): The square root of the mean of the squared deviations, reflecting overall surface discrepancy.
» Positive Average (+Avg): The mean of all positive deviation values.
» Negative Average (−Avg): The mean of all negative deviation values.

To assess variability introduced by the scanning process itself, the reference model was scanned 15 times under identical conditions, and the resulting datasets were compared to determine scan-induced deviation.

To assess intra-examiner reliability, 20 models were randomly selected and rescanned by the same operator one week after the initial scan. Root mean square (RMS) deviation values were calculated from the repeated scans, and statistical comparison was used to evaluate reproducibility. 

## STATISTICAL ANALYSIS

All statistical analyses were performed using SPSS software (version 22.0; IBM Corp., Armonk, NY, USA). Descriptive statistics, including means and standard deviations, were calculated for each group. Intra-examiner reliability was assessed using the intraclass correlation coefficient (ICC), based on a two-way mixed-effects model with absolute agreement.

The normality of data distribution was verified using the Shapiro-Wilk test. Since the data followed a normal distribution, one-way analysis of variance (ANOVA) was applied to evaluate intergroup differences. When significant differences were detected, pairwise comparisons were conducted using Tukey’s Honestly Significant Difference (HSD) *post-hoc* test. A significance level of *p* < 0.05 was adopted for all analyses. 

## RESULTS

### INTRA-EXAMINER RELIABILITY

The ICC value for repeated RMS measurements was 0.931, indicating excellent intra-examiner reliability.

### COMPARISON OF DIASTEMA GROUPS

Statistically significant differences were found among the diastema groups for all evaluated parameters (*p* < 0.05). The 5 mm diastema group exhibited the greatest overall deviation, with a minimum of −0.613 ± 0.009 mm and a maximum of 0.619 ± 0.012 mm.

Both the 2.5 mm and 5 mm diastema groups showed significantly higher RMS, +Avg, and −Avg values, compared to the control model. In the 2.5 mm group, RMS was 0.114 ± 0.005 mm, +Avg was 0.089 ± 0.005 mm, and −Avg was −0.086 ± 0.005 mm. These values were more pronounced in the 5 mm group (RMS: 0.128 ± 0.004 mm; +Avg: 0.100 ± 0.008 mm; −Avg: −0.103 ± 0.007 mm). In contrast, the 10 mm diastema group demonstrated lower deviation values (RMS: 0.095 ± 0.014 mm; +Avg: 0.077 ± 0.017 mm; −Avg: −0.083 ± 0.010 mm).

These findings suggest that smaller diastemas (≤5 mm) may lead to greater dimensional inaccuracies in 3D-printed models, potentially due to increased difficulty in scan capture caused by narrow spacing. Conversely, wider diastemas (10 mm) may enhance scan accessibility, thereby reducing surface deviations (Table 1). 

**Table 1:** Comparison results between diastema models.

Parameters	Min	Max	RMS	+ Average	- Average
	Mean ± SD	Mean ± SD	Mean ± SD	Mean ± SD	Mean ± SD
Reference (Original Digital Model)	-0.302 ± 0.011^A^	0.339 ± 0.367^A^	0.083 ± 0.008^A^	0.063 ± 0.006^A^	-0.071 ± 0.008^A^
2.5 mm diastema	-0.485 ± 0.022^B^	0.496 ± 0.012^B^	0,114 ± 0.005^BC^	0.089 ± 0.005B^C^	-0.086 ± 0.005B^C^
5 mm diastema	-0.613 ± 0,009^C^	0.619 ± 0.012^C^	0.128 ± 0.004^C^	0.100 ± 0.008^C^	-0.103 ± 0.007^C^
10 mm diastema	-0.266 ± 0.024^A^	0.332 ± 0.038^A^	0.095 ± 0.014^AB^	0.077 ± 0.017^AB^	-0.083 ± 0.010^AB^
p value	<0.001^*^	<0.001^*^	<0.001^*^	0.002^*^	0.001^*^

Comparison of the measurements with one-way ANOVA test between the groups. Different capital letters denote statistical differences between groups within the same column. Each measurement is reported in millimeters. Min = The greatest negative deviation value, indicating areas that are positioned negatively in relation to the reference model. Max = The greatest positive deviation value, indicating areas that are positioned positively in relation to the reference model. RMS = The square root of the arithmetic mean of the squares of the deviation values. + Average = The average of all positive deviations, indicating areas that are positioned positively in relation to the reference model. - Average = The average of all negative deviations, indicating areas that are positioned negatively in relation to the reference model. SD: standard deviation, *p < 0.05.

### COMPARISON OF CROWDING GROUPS

Significant differences were observed among the crowding groups in terms of minimum deviation values (*p* < 0.001). The lowest minimum value (−0.368 ± 0.006 mm) was recorded in the very severe crowding group (12 mm), which also exhibited the highest maximum deviation (0.378 ± 0.006 mm) (p < 0.008).

Although RMS and +Avg values did not differ significantly across all groups, the 12 mm crowding group demonstrated the highest values for both parameters (RMS: 0.091 ± 0.003 mm; +Avg: 0.071 ± 0.001 mm). The difference in +Avg was borderline significant (p = 0.053). No statistically significant differences were detected among the groups for - Avg values (p = 0.260).

Overall, these results indicate that increased severity of anterior crowding - particularly in the 12 mm group - adversely affects the dimensional accuracy of 3D-printed models, as evidenced by elevated Min, Max, RMS, and +Avg values (Table 2). 

**Table 2:** Comparison results between crowding models.

Group	Min	Max	RMS	+ Average	- Average
	Mean ± SD	Mean ± SD	Mean ± SD	Mean ± SD	Mean ± SD
Reference (Original Digital Model)	-0.302 ± 0.011^BC^	0.339 ± 0.367^AB^	0.083 ± 0.008^A^	0.063 ± 0.006^AB^	-0.071 ± 0.008^A^
Minimum crowding (3 mm)	-0.273 ± 0.186^C^	0.317 ± 0.020^A^	0.079 ± 0.006^A^	0.060 ± 0.005^A^	-0.069 ± 0.006^A^
Moderate crowding (6mm)	-0.310 ± 0.039^BC^	0.319 ± 0.030^A^	0.081 ± 0.006^A^	0.062 ± 0.004^AB^	-0.067 ± 0.005^A^
Severe crowding (9 mm)	-0.344 ± 0.019^AB^	0.369 ± 0.016^AB^	0.085 ± 0.005^A^	0.067 ± 0.004^AB^	-0.070 ± 0.005^A^
Very severe crowding (12 mm)	-0.368 ± 0.006^A^	0.378 ± 0.006^B^	0.091 ± 0.003^A^	0.071 ± 0.001^B^	-0.078 ± 0.005^A^
p value	<0.001^*^	0.008^*^	0.118^*^	0.053^*^	0.260^*^

Comparison of the measurements with one-way ANOVA test between the groups. Different capital letters denote statistical differences between groups within the same column. Each measurement is reported in millimeters. Min = The greatest negative deviation value, indicating areas that are positioned negatively in relation to the reference model. Max = The greatest positive deviation value, indicating areas that are positioned positively in relation to the reference model. RMS = The square root of the arithmetic mean of the squares of the deviation values. + Average = The average of all positive deviations, indicating areas that are positioned positively in relation to the reference model. - Average = The average of all negative deviations, indicating areas that are positioned negatively in relation to the reference model. SD: standard deviation, *p < 0.05.

## DISCUSSION

This study investigated the influence of varying severities of anterior crowding and diastema on the dimensional accuracy of 3D-printed dental models. Although the clinical significance of 3D printing accuracy is well established, limited attention has been paid in the literature to how intra-arch anatomical variations - such as spacing and crowding - affect the fidelity of printed models.^6^ ^,^ ^13^ ^,^ ^14^ ^,^ ^17^ Moreover, many existing studies are constrained by methodological limitations, including the lack of standardized classifications for crowding and diastema severity, and inadequate control over scanning-related variables. The present study addressed these issues through a controlled experimental design involving clearly defined, reproducible anterior modifications and a region-specific superimposition protocol.

For instance, Grassia et al.^6^ examined the effects of anatomical complexity by printing digital models of two patients - one presenting with anterior crowding, and the other with a missing posterior tooth - ten times, using four different 3D printers. While their study focused on comparing printer-related accuracy, it did not stratify the degree of crowding or spacing, leaving it unclear whether observed deviations were attributable to printer characteristics, anatomical morphology, or inconsistencies in the scanning process.

Similarly, Spangler et al.^14^ evaluated surface deviations in aligners fabricated for thirty patients with three levels of crowding, using both 3D printing and vacuum forming techniques. However, deviations were analyzed on the aligners themselves, rather than on the digital or printed models, making it difficult to distinguish whether dimensional inaccuracies originated from the printing process or from aligner fabrication.

Koenig et al.^18^ also compared the accuracy of directly printed versus thermoformed aligners using a single reference model and two thermoplastic materials. Their focus was primarily on the influence of manufacturing techniques and material properties, without assessing the potential role of anatomical variation in model precision.

Another frequent limitation in prior studies is the reliance on full-arch superimposition, which may obscure region-specific deviations by averaging surface discrepancies across both altered and unaltered areas.^14^ ^,^ ^17^ In contrast, the present study employed a localized superimposition strategy, by confining morphological changes to the anterior region and using unmodified posterior teeth as stable reference landmarks. This approach enabled precise quantification of deviations specifically within the area affected by crowding and diastema.

Several studies have reported that extrinsic variables - such as salivary contamination, ambient lighting, and scanning trajectory - can influence the accuracy of intraoral scans.^19^ ^,^ ^20^ To minimize the potential effects of these confounding factors, strict standardization protocols were implemented in the present study. All scans were performed by a single experienced operator, and the scanner was recalibrated prior to each session. Additionally, all scans were conducted at consistent times of day, and ambient light intensity was monitored using a luxmeter, to ensure uniform illumination.^21^

**Figure 4:**
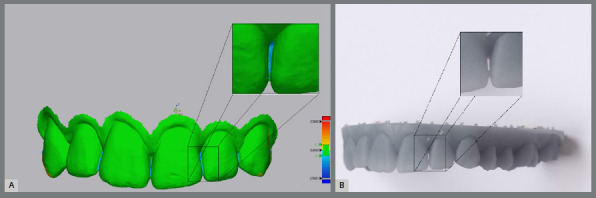
A) Surface deviation seen after superimposition in the diastema region, B) Error areas noticed with the naked eye in the diastema regions.

To quantify the variability introduced by the scanning procedure itself, the primary 3D-printed reference model was scanned 15 times under identical conditions. Superimposition of these scans revealed minimal surface deviations, with a +Avg of 0.0032 mm and a − Avg of −0.0014 mm.^22^ These findings suggest that the intraoral scanning process contributed negligibly to the overall surface deviation, thereby reinforcing the reliability of the digital acquisition protocol (Fig. 4).

To further verify the dimensional precision of the 3D printer used in this study, the +Avg values were compared with those reported in previous investigations with similar aims. For instance, Koenig et al.^18^ and others^23^ documented +Avg values ranging from 0.079 mm to 0.224 mm. In comparison, the present study demonstrated +Avg values ranging from 0.060 ± 0.005 mm to 0.100 ± 0.008 mm, indicating that the printer employed in this study exhibited superior precision.

Another factor known to affect print accuracy is layer thickness, which influences surface smoothness and anatomical detail resolution. Although several studies have concluded that a thickness of 100 µm is adequate for dental model fabrication, the present study utilized a finer resolution of 50 µm, to enhance the dimensional fidelity of the printed models.^24^ ^,^ ^25^

A notable strength of this study lies in the comprehensive evaluation of surface deviation metrics. Whereas most prior investigations have focused exclusively on RMS values or average deviations (±Avg),^6^ ^,^ ^13^ ^,^ ^18^ the current analysis also incorporated Min and Max deviation values. This expanded approach enabled a more thorough characterization of surface discrepancies and improved the interpretive depth of the findings.

While the present results align with those of Grassia et al.^6^ in suggesting that crowding does not significantly affect overall model accuracy, the present study found that diastemas of 2.5 mm and 5 mm led to significantly greater surface deviations. Notably, the study by Grassia et al.^6^ did not standardize the severity of crowding or spacing, and focused primarily on extraction spaces rather than true anterior diastemas. In contrast, the present study implemented precise and reproducible modifications for both crowding and diastema, enabling a more accurate and isolated assessment of their respective effects on 3D printing accuracy.

The findings of this study demonstrate that minor anterior diastemas and severe dental crowding negatively affect the dimensional accuracy of 3D-printed orthodontic models. Clinically, this has important implications, particularly in clear aligner therapy, where treatment planning and appliance fabrication rely heavily on the accuracy of prototype models. Inaccurate replication of interproximal surfaces may compromise the precision of programmed tooth movements, especially those related to space closure and alignment.

Cases involving small diastemas - particularly those under 1 mm - were associated with statistically significant deviations. In such scenarios, even subtle geometric discrepancies can disrupt force application or alter interproximal contact relationships during aligner wear. Consequently, residual spacing or incomplete tooth movement may persist at the end of treatment, potentially necessitating additional refinement phases, thereby increasing overall treatment duration and cost.

Given these results, clinicians are advised to exercise increased vigilance when managing cases involving minor spacing or pronounced anterior crowding. Awareness of the potential for reduced model fidelity in these situations may support earlier intervention strategies, more frequent monitoring, or preemptive refinement planning. Incorporating this understanding into clinical workflows may enhance treatment predictability and reduce the likelihood of mid-course corrections.

Despite the methodological strengths of this investigation - including standardized manipulation of anterior spacing and crowding, and the use of region-specific superimposition techniques -, several limitations should be acknowledged. The study utilized a single intraoral scanner and 3D printer, and its analysis was confined to the anterior segment. As such, the generalizability of the findings to other scanner-printer combinations or to different anatomical regions (e.g., posterior teeth) may be limited. Future studies incorporating multiple scanning and printing systems, as well as broader dental arch segments, are warranted to validate and extend these results.

## CONCLUSION

The null hypothesis was partially rejected. The results demonstrated that very severe anterior crowding (12 mm) and minimal diastemas (<1 mm between adjacent teeth) significantly compromise the dimensional accuracy of 3D-printed orthodontic models. These findings highlight the importance of accounting for intra-arch anatomical variations - particularly minimal spacing and pronounced crowding - during digital model acquisition and fabrication. Clinicians should consider these factors when planning clear aligner therapy, in order to improve appliance precision, enhance treatment efficiency, and achieve more predictable clinical outcomes.

## Data Availability

Data and materials are available at the Orthodontic Department in the Faculty of Dentistry, University of Afyonkarahisar Health Sciences University.
